# On the role of dauer in the adaptation of nematodes to a parasitic lifestyle

**DOI:** 10.1186/s13071-021-04953-6

**Published:** 2021-10-27

**Authors:** Lieke E. Vlaar, Andre Bertran, Mehran Rahimi, Lemeng Dong, Jan E. Kammenga, Johannes Helder, Aska Goverse, Harro J. Bouwmeester

**Affiliations:** 1 Plant Hormone Biology Group, Green Life Sciences Cluster, Swammerdam Institute for Life Sciences, University of Amsterdam, Science Park 904, 1098 XH Amsterdam, The Netherlands; 2 Laboratory of Nematology, Department of Plant Sciences, Wageningen University, 6708 PB Wageningen, The Netherlands

**Keywords:** Dauer, Parasitic nematodes, Clade 12, Quiescence, *Globodera*

## Abstract

Nematodes are presumably the most abundant Metazoa on Earth, and can even be found in some of the most hostile environments of our planet. Various types of hypobiosis evolved to adapt their life cycles to such harsh environmental conditions. The five most distal major clades of the phylum Nematoda (Clades 8–12), formerly referred to as the Secernentea, contain many economically relevant parasitic nematodes. In this group, a special type of hypobiosis, dauer, has evolved. The dauer signalling pathway, which culminates in the biosynthesis of dafachronic acid (DA), is intensively studied in the free-living nematode *Caenorhabditis elegans*, and it has been hypothesized that the dauer stage may have been a prerequisite for the evolution of a wide range of parasitic lifestyles among other nematode species. Biosynthesis of DA is not specific for hypobiosis, but if it results in exit of the hypobiotic state, it is one of the main criteria to define certain behaviour as dauer. Within Clades 9 and 10, the involvement of DA has been validated experimentally, and dauer is therefore generally accepted to occur in those clades. However, for other clades, such as Clade 12, this has hardly been explored. In this review, we provide clarity on the nomenclature associated with hypobiosis and dauer across different nematological subfields. We discuss evidence for dauer-like stages in Clades 8 to 12 and support this with a meta-analysis of available genomic data. Furthermore, we discuss indications for a simplified dauer signalling pathway in parasitic nematodes. Finally, we zoom in on the host cues that induce exit from the hypobiotic stage and introduce two hypotheses on how these signals might feed into the dauer signalling pathway for plant-parasitic nematodes. With this work, we contribute to the deeper understanding of the molecular mechanisms underlying hypobiosis in parasitic nematodes. Based on this, novel strategies for the control of parasitic nematodes can be developed.

**Supplementary Information:**

The online version contains supplementary material available at 10.1186/s13071-021-04953-6.

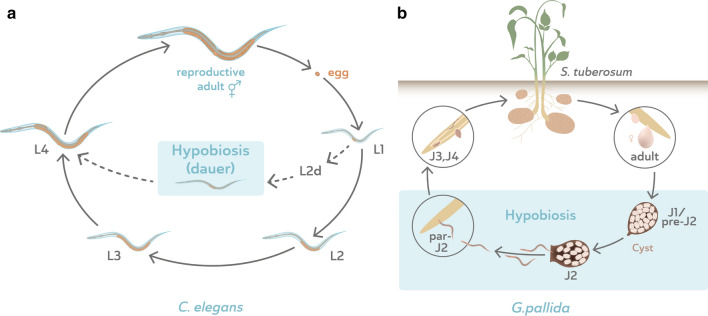

## Background

Nematodes, also referred to as roundworms, play an important role in many ecosystems, from ocean floors to arable soils. Stemming from a common non-parasitic ancestor, most contemporary nematodes are free-living and trophically diverse. As such, they are represented in all major trophic levels in soil and sediment food webs. Nematodes can be subdivided based on their trophic behaviour. According to this criterion, they can be divided into three groups: free-living, obligate parasitic and facultative parasitic nematodes. The first feed predominantly on microorganisms such as bacteria or fungi and, in the case of predatory free-living nematodes, they devour other nematode species. Obligate parasitic nematodes feed on plant, invertebrate or vertebrate hosts. Facultative parasitic nematodes are usually more flexible in their food source; in a free-living cycle they feed on microbes, whereas in a parasitic cycle they parasitize a host organism [1]. Interestingly, parasitism has evolved at least 18 times independently within the phylum Nematoda [2, 3].

It is estimated that the cost of parasitic diseases caused by nematodes in livestock worldwide reaches into the tens of billions of US dollars annually [4]. In addition, six of the 13 core neglected tropical diseases in humans are caused by nematodes [5]. It is noted that only a numerically very limited subset of these parasitic nematodes is responsible for this damage (e.g. [2]). Control of these nematodes is difficult, and many anthelmintic drugs have become ineffective due to natural selection for resistant populations [6]. In agriculture, plant-parasitic nematodes cause over US$ 100 billion of losses annually [7]. Until recently, fumigants (general biocides) were massively applied to control plant parasites. They are now largely banned because of their non-specificity and their high toxicity. Hence, a relatively small fraction of parasitic nematodes pose a huge burden on humans, both in terms of health and food safety, and socio-economically.

In order to develop new tools for the control of parasitic nematodes, in humans and animals as well as in agriculture, a better understanding of the mechanisms underlying the parasitic lifestyle is needed. The phylum Nematoda is divided into 12 clades, of which the common ancestor is considered non-parasitic. Up to now, parasitism has been found in Clades 1, 2, 5, and 8 to 12 [2, 3, 8, 9]. For the evolution of parasitism in Clades 9 and 10, it has been hypothesized that a specific type of hypobiosis, called dauer, is critical because it enables the nematode to persist in the temporary absence of food [10]. Specific for dauer is the dauer signalling pathway, which underlies dauer of nematodes in Clades 9 and 10. In this review, we investigate the hypothesis that the dauer pathway was also essential for the evolution of parasitism in Clade 12. To do so, a hybrid definition of dauer is used as a state in which the larva is (i) non-feeding and (ii) essentially non-ageing, and the species has (iii) a conserved dafachronic acid biosynthetic pathway that produces (a chemical analogue of) dafachronic acid. Additional morphological characteristics, such as radial constriction and/or a sealed buccal capsule, the ability to survive some degree of desiccation and the ability to adopt a coiled posture, are characteristic for dauer in some species, but are not ubiquitous. Finally, we discuss the literature on the perception of host cues and their probable integration into the dauer signalling pathway.

## Hypobiosis in nematodes

**Fig. 1:**
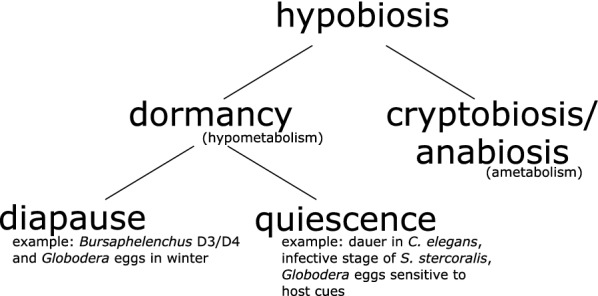
Hypobiosis can be divided into cryptobiosis and dormancy. The latter can in turn be divided in diapause and quiescence. An important example of quiescence is dauer in *Caenorhabditis elegans* and its related stages in parasitic nematodes

Hypobiosis, or developmental arrest, is a state of reduced metabolic activity and exists in two different forms: cryptobiosis and dormancy (Fig. 1). Cryptobiosis, or anabiosis, is more extreme in that organisms in this state show almost no sign of life. Anhydrobiosis is a rare form of cryptobiosis, in which a nematode species can survive without water. Dormancy, on the other hand, is a fairly ubiquitous phenomenon among nematodes. Dormancy comprises two related but non-identical states: diapause and quiescence [11].

Organisms in diapause are dormant according to season, under influence of temperature, and remain in this state for a set time, even if favourable circumstances return [12]. An example of diapause in nematodes are the *Bursaphelenchus xylophilus* dispersal stage 3 and 4 (D3, D4) juveniles that stay dormant in winter [13]. Similar features are seen in cyst nematodes of the genera *Globodera* and *Heterodera*; pre-parasitic second stage juveniles (pre-J2) go into a seasonal diapause in winter or summer, while still inside the egg [14, 15]. While in diapause, they are insensitive to external host cues that otherwise would induce hatching. Diapause might be lifted by a certain temperature sum, for example, or rising spring temperature. Once diapause is lifted by the increasing temperatures of spring, they are perceptive to hatching stimuli again. Until they perceive these host-derived stimuli, they remain quiescent. A quiescent organism responds to abiotic or biotic cues in order to synchronize its development with its environment [11]. The best-studied form of quiescence in nematodes is dauer, first identified in *C. elegans* [16]. Moreover, quiescence is widely spread across zooparasitic nematodes, such as *Strongyloides* spp., and is exited upon perception of a host cue. In general, parasitic nematode species may enter diapause or quiescence, and often they can do both.

### The dauer stage

**Fig. 2:**
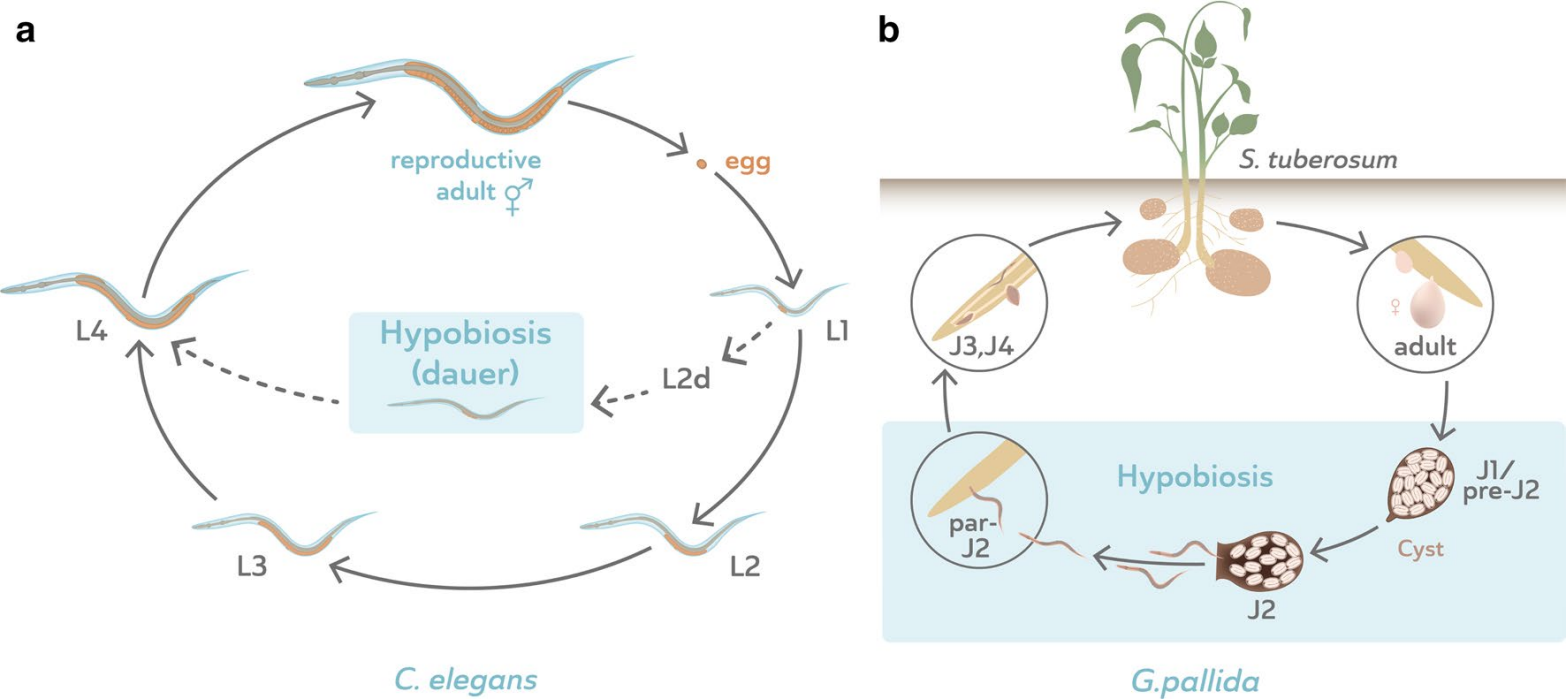
The life cycles of the free-living model nematode *Caenorhabditis elegans* (**a**) and the parasitic potato cyst nematode *Globodera pallida* (**b**). The hypobiotic stage in each species is indicated with a blue box. L1, L2, L3, L4 = larval stage 1, 2, 3 and 4. J1, J2, J3, J4 = juvenile stage 1, 2, 3 and 4. Pre-J2 = pre-parasitic juvenile stage 2. Par-J2 = parasitic juvenile stage 2

All nematodes go through four larval stages, which are called L1 to L4 (larval stage 1 to 4) for free-living and zooparasitic nematodes, and J1 to J4 (juvenile stage 1 to 4) for entomopathogenic and plant-parasitic nematodes. Dormancy may occur in distinct life stages in a species-dependent manner. The most thoroughly studied form of dormancy is dauer (from the German ‘enduring’) in *C. elegans*, which is an alternative L3 stage (Fig. 2a), only entered upon harsh conditions such as crowding, lack of food or high temperature. In dauer, *C. elegans* displays a distinct morphology: it shows radial constriction, has alae (longitudinal ridges in the cuticle), a sealed buccal plug that does not allow feeding, and a double cuticle which ensures resistance to stresses such as desiccation and toxic chemicals [10, 16]. Moreover, *C. elegans* dauers rely on storage lipids as a source of energy, as evident from the increased activity of enzymes in the glyoxylate pathway in dauer worms compared to other developmental stages [17–19]. The developmental decision to enter dauer is made 15 to 33 h after hatching [20] depending on the abovementioned environmental conditions. If these are favourable, the worm develops into L3, L4 and reproductive stages, but if not, it enters the pre-dauer state L2d and subsequently dauer. The dauer stage can last for months, and only when ambient conditions improve will the worms exit dauer and resume reproductive development. Dauer is also a dispersal stage with accompanying behaviour, called nictation, a movement in which the worm stands on its tail and waves its upper body, trying to attach to a vector in order to hitchhike to a new environment [21]. Additionally, the dauer worm is attracted to CO_2_, which facilitates the detection of potential vectors [22].

Dauer occurs in all members of the genus *Caenorhabditis*, as well as in other closely related free-living nematodes such as *Pristionchus pacificus*. Sudhaus [10] defined the dauer larva as ‘an alternative third stage juvenile which does not feed, has a closed stoma and retains the moulted cuticle of the previous stage as a protective sheath’. Many parasitic nematodes have an infective stage that resembles the *C. elegans* dauer. Riddle [23] wrote in *C. elegans* II that ‘it seems likely that diverse parasites will have used similar (to the basic signal transduction machinery in *C. elegans*) internal pathways linked to different cues’, in order to incorporate host cues into the regulation of dauer entry and exit. Whereas in some parasitic nematodes dauer is an alternative life stage only entered upon certain (unfavourable) conditions, in others it is an obligate stage that will be entered during every cycle, regardless of ambient conditions (Fig. 2).

**Fig. 3:**
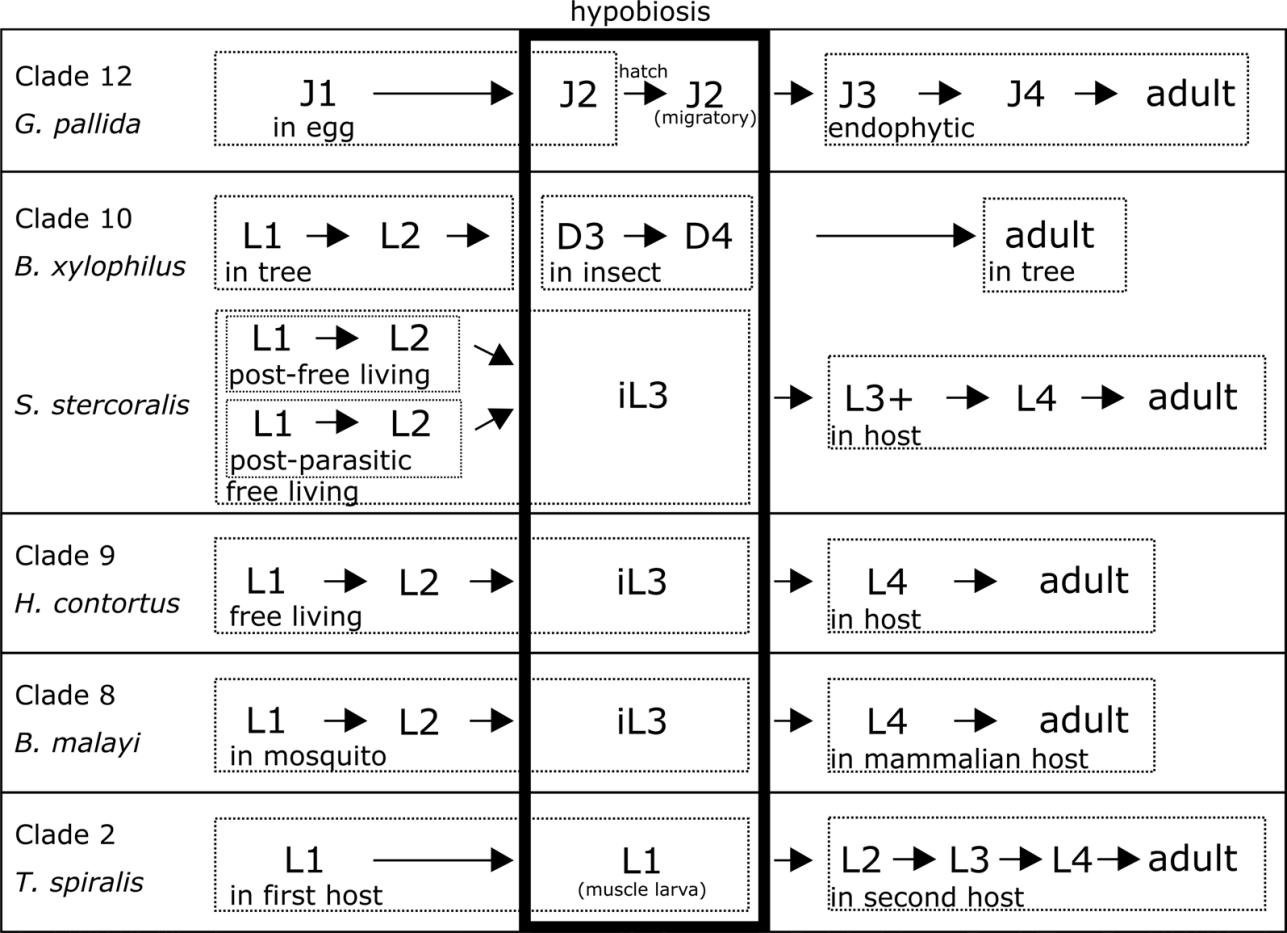
Schematic overview of life cycles of representatives of parasitic nematode of clades 2, 8, 9, 10 and 12. The hypobiotic stage is marked with a box. J1, J2, J3, J4 = juvenile stage 1, 2, 3 and 4. L1, L2, L3, L4 = larval stage 1, 2, 3 and 4. iL3 = infective larval stage 3. L3+ = post-infective third larval stage. D3, D4 = dauer stage 3 and 4

Curiously, nematologists from different fields use different definitions. Zooparasitic scientists use a strict definition that approaches that of Sudhaus and does not consider the infective larval stage of a zooparasite to be a dauer stage, although the helminth community acknowledges that the infective larval stage probably arose from the dauer stage [24]. In plant-parasitic nematology, a wider definition of dauer is used which does not distinguish between the equivalent of the *C. elegans* dauer stage and the infective stage of parasitic nematodes (ref. [25] and Figs. 2, 3). The consequence is that dauer is difficult to define exactly and is actually a collection of stages of quiescence, having in common the absence of ageing and feeding and, as we will explain in the next paragraph, the probable conservation of genes of a specific part of the dauer signalling pathway.

### The dauer signalling pathway

**Fig. 4:**
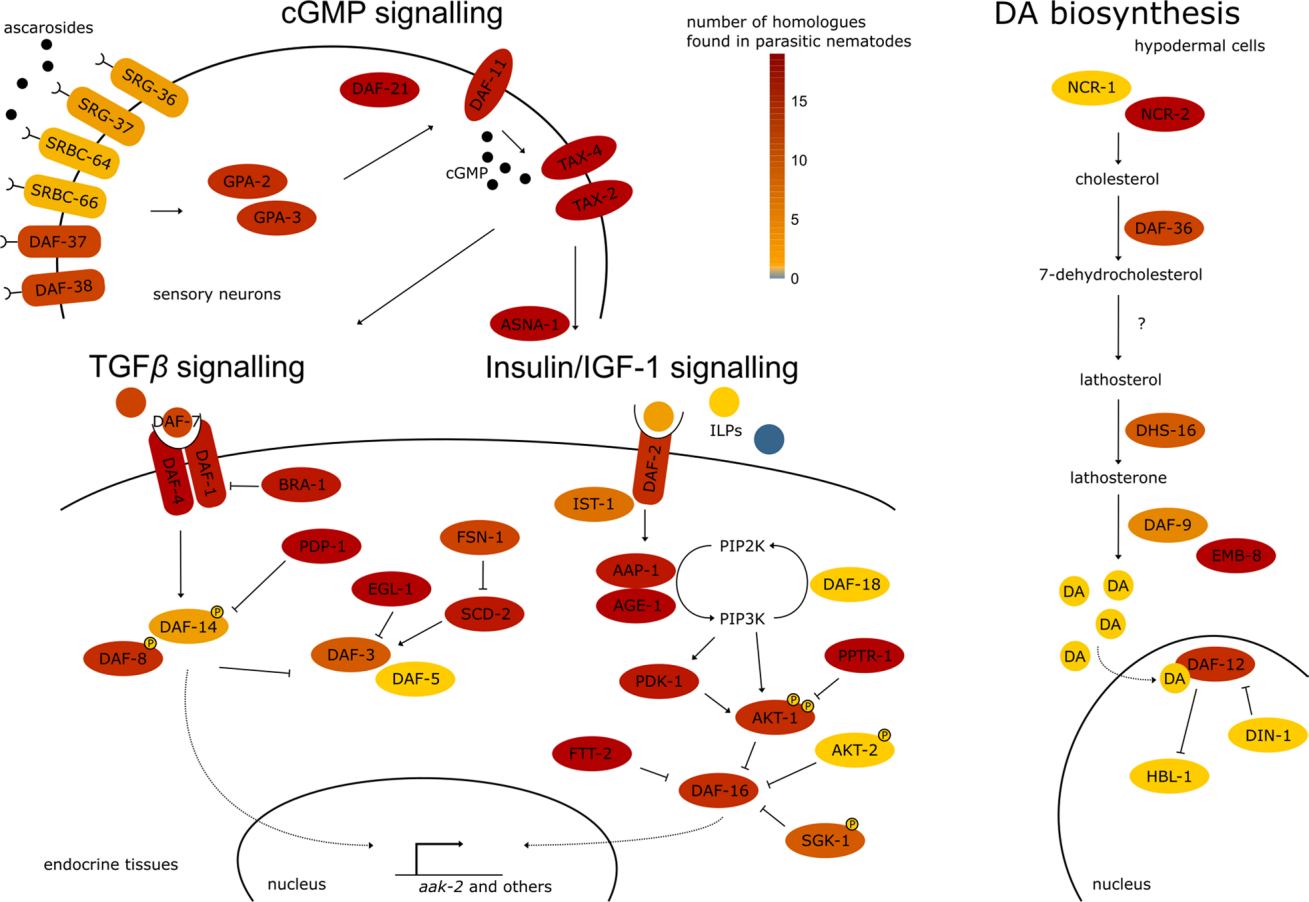
Schematic overview of the dauer signalling pathway in *Caenorhabditis elegans* consisting of the cGMP signalling pathway, the parallel TGF-β and insulin/IGF-1 pathways, and the concluding DA biosynthetic pathway. The colour scale indicates how conserved each gene is across parasitic nematodes: blue is not conserved, yellow towards red indicates that a gene is less or more conserved. This figure was redrawn from references [1, 166]

The dauer signalling pathway has been thoroughly studied, mainly because of its role in ageing [26] and the analogy of dauer with the infective stages of zooparasitic nematodes causing disease in humans and animals [24]. Roughly, this pathway consists of four sub-pathways: the cyclic guanosine monophosphate (cGMP) signalling pathway, which feeds into the parallel transforming growth factor beta (TGF-β) and insulin/insulin-like growth factor 1 (IGF-1) pathways, which in turn feed into the dafachronic acid (DA) biosynthesis pathway (Box 1, Fig. 4). The first three are conserved throughout most of the animal kingdom; the latter is specific to the phylum Nematoda.

In the second messenger system, recognition of a ligand by a G-protein-coupled receptor (GPCR) results in the production of a secondary messenger such as cGMP. This is a ubiquitous system for signal transduction found in many different cell types, not only in animals, but also plants and fungi. It allows for extracellular signals to be communicated towards an intracellular response, for example in the case of perception of hormones. The *C. elegans* genome encodes more than 1000 *gpcr* genes, of which most are nematode-specific [27], and *gpcr* genes have also been identified in other nematodes [28]. They play roles in chemotaxis and more specifically, for parasitic nematodes, in host detection [29–31]. The TGF-β signalling pathway is involved in multiple cellular processes in animals, such as development, differentiation, immune reaction and growth [32]. In *C. elegans*, this pathway regulates, apart from dauer, body size, axon pathfinding and male copulatory organ development [33]. Also, the insulin/IGF-1 pathway is found throughout the animal kingdom and has been shown to be involved in ageing [34]. Thus, these three sub-pathways are not singularly linked to dauer in nematodes, but the pathway they converge on, the DA signalling pathway, is. DA is synthesized from cholesterol by multiple enzymes, of which some are as yet unknown. The last biosynthetic step is executed by DAF-9, a cytochrome P450 (CYP450) enzyme [35, 36]. This results in the formation of DA, that binds to the nuclear receptor DAF-12, which prevents dauer entry or results in dauer exit (ref. [37, 38] and Fig. 4). Although DA was detected in all life stages, a 30-fold increase in its concentration is measured upon dauer evasion [39]. Since the DA biosynthesis pathway has been shown to be specifically responsible for the regulation of dauer, we postulate that the conservation of this pathway, and hence a peak in (chemical analogue of) DA biosynthesis upon exit of a hypobiotic stage, is a prerequisite to classify a quiescent stage as dauer, in addition to the absence of ageing and feeding that were mentioned earlier.

### The pre-adaptation concept

**Fig. 5:**
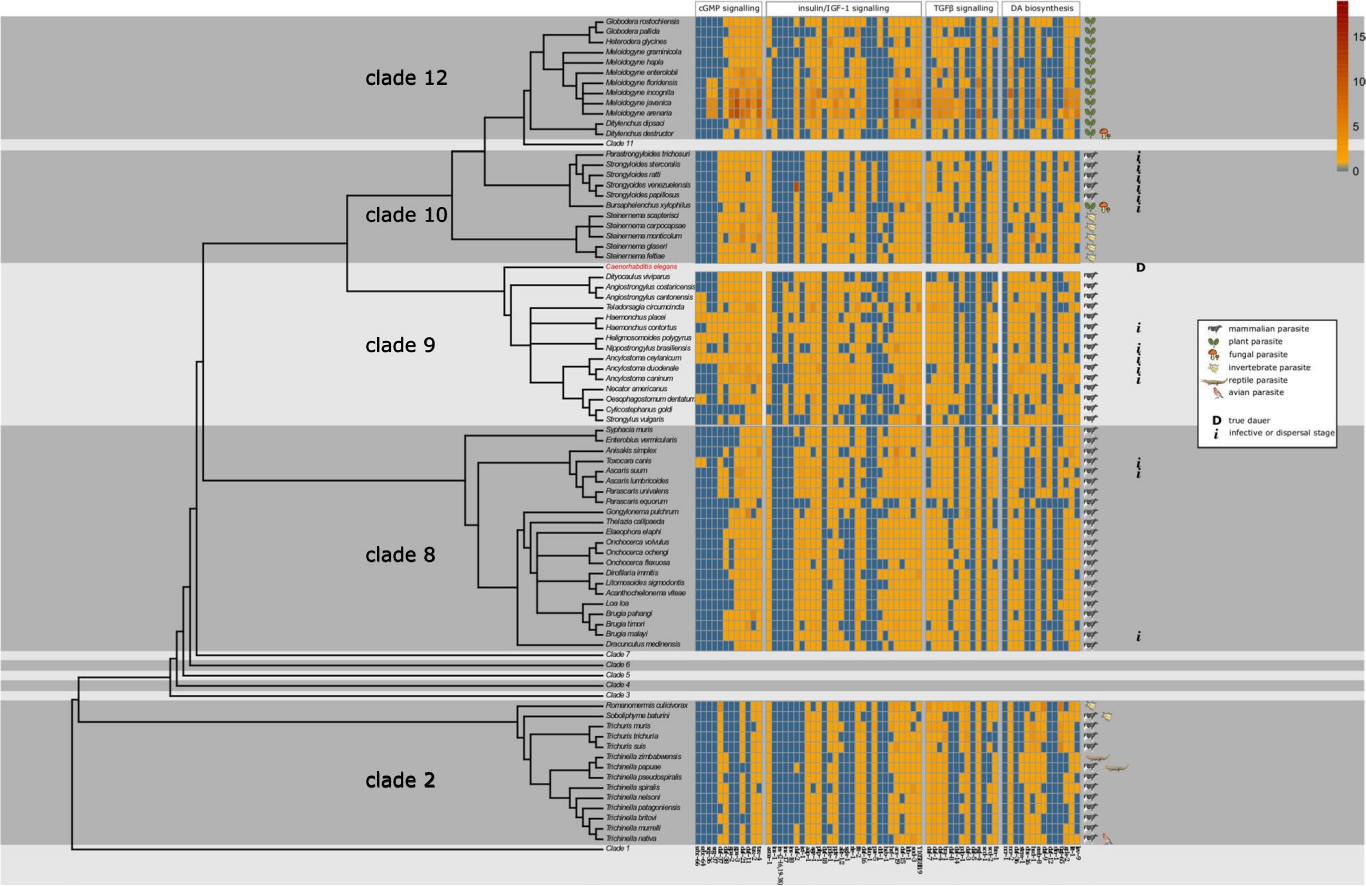
Phylogenetic tree of *Caenorhabditis elegans* and parasitic nematodes of which the genome was sequenced and a heat map showing which genes of the dauer signalling pathway are conserved. The type of parasitism each species displays is indicated, as well as whether they have a life cycle with a dauer (only *C. elegans*) or a confirmed dauer-derived infective stage. The same colour scale as in Fig. 4 was used, blue indicating no homologue, and yellow towards red indicating one or more homologues were detected. In order to assemble this information, we used the WormBase ParaSite (release number 14.0) BioMart orthologue finder tool on a comprehensive list of dauer-related genes and queried all available genomes of parasitic nematodes available in this database (ref. [85], parasite.wormbase.org) using the ‘gene stable ID’, ‘gene name’, ‘homology type’, and the two ‘% identity options’ within the Orthologues tab in ‘Output Attributes’. BioMart orthologue finder tool outputs were generated individually for each *C. elegans*-to-parasitic species comparison. One-to-one orthologues were taken directly from the output files into the matrix used to prepare the figure. For all other homology types, the most likely orthologues in the parasitic species were chosen by the highest pair of % identity values. Accession numbers are retrievable in Additional file 1: Table S1

The phylum Nematoda is divided into 12 clades, and the greatest diversity in parasitic lifestyles is found in Clades 8 to 12, formerly referred to as the Secernentea (Fig. 5 and ref. [40]). Additionally, parasitic nematodes are present in Clades 1 and 2. Clade 9 harbours many bacterivorous species with a preference for saprobious habitats, which favour the development of parasitism, according to the pre-adaptation concept described by Sudhaus [10]. In short, basal members of Clade 9—mainly Rhabditidae—live in dead and decaying organic matter, which are isolated sources of bacterial food that may be decomposed and are exhausted in a short period of time—the so-called cycles of boom and bust [41]. To adapt successfully to a zooparasitic lifestyle, these nematodes evolved the following characteristics: (i) the capacity to survive under low oxygen levels, (ii) the capacity to withstand varying osmotic pressures, (iii) a tolerance to digestive enzymes produced by bacterial and fungal decomposers, (iv) a tolerance to high temperatures, (v) increased reproductive capacity, and (vi) the capacity to use vectors to disperse among isolated habitats and (vii) to redirect or arrest development to prevent complete exhaustion of reserves until a new food source has been found [10]. The latter two of these pre-adaptations require the nematode to redirect its development into an enduring stage, in order to survive for a long time under unfavourable conditions. Here, too, nematodes in this stage are devoid of an external food source and hence will lower their metabolism. Furthermore, they need to withstand adverse environments while at the same time trying to localize and enter a vector, usually an insect, to be transported to a new food source, where they can resume development. Hostile conditions are common, and it is expected that free-living nematode species, that can enter dauer, spend most of their lifetime in this state [42].

For the evolution of animal (invertebrate and vertebrate) parasitism in Clade 9, the seven pre-adaptations described by Sudhaus [10] are essential. There are several intermediate life forms between free-living and parasitic nematodes, and it is assumed that via these, parasitism in this clade has evolved. From the above-described phoretic relationship, in which nematodes travel to new habitats using insect vectors to which they do not inflict any harm, nematodes developed a lifestyle called necromeny [10]. This is a commensalistic symbiosis in which one organism benefits greatly, whereas the other is unaffected, a lifestyle common in *Pristionchus* species [37]. In necromeny, nematodes remain in a developmental arrest in the insect without derogating the latter, until the insect dies, after which the nematodes start feeding on the bacterial community that invades the insect corpse [10]. The next evolutionary step towards parasitism is entomoparasitoidy, occurring for example in the Heterorhabditidae, in which the nematode reproduces inside the insect and the insect is killed by nematode-associated microbes. Offspring can then feed on a pre-digested nutrient broth in the insect corpse, which disqualifies this lifestyle as truly parasitic, since the nematode itself does not inflict harm onto the insect, but instead, the associated microbes do [10]. Nevertheless, from here, the step towards true parasitism is small. Outside Clade 9, facultative parasitism occurs, in which the nematode retains the option to complete its life cycle independent of a host, while, if a host is available, the nematode will take advantage and parasitize [38]. By contrast, obligate parasites are fully dependent on the availability of their host; if available, they proceed with reproductive development for multiple generations and thereby quickly colonize their habitat—for example, small ruminants in the case of the parasite *Haemonchus contortus* [43]. If a host is not available, some of the Clade 9 species can stay dormant for months [16], only to resume reproductive development as soon as a host reappears (Fig. 3).

### The dauer hypothesis

Dauer-like features are observed in both free-living and parasitic nematodes throughout Clades 8 to 12, even though their life cycles differ substantially (Figs. 2, 3). Parasites need to adjust their life cycle to that of their hosts in order to ensure food availability by efficiently infecting them. Quiescent stages are a means to achieve synchronization, and these are analogous to the dauer in *C. elegans* [41, 44], which is a facultative third larval (L3) stage. In Clades 8, 10 and 12, quiescent juveniles display more deviations from dauer, although they still have one or more of the following features: radial constriction, a thickened cuticle, sealed buccal capsule and non-feeding behaviour.

The hypothesis that dauer is one of the prerequisites for the evolution of parasitism in Clade 9 is formulated in ‘the dauer hypothesis’ [10, 24, 41, 44]. However, this hypothesis has largely focused on zooparasites of Clades 8, 9 and 10, whereas indications for a dauer state also exist in Clade 12. Moreover, this concept is probably true for the Strongylida of Clade 9, which presumably arose from bacterivorous rhabditids. Yet, there is no phylogenetic evidence that parasitism has evolved in a similar way in Clade 8, since a bacterivorous ancestor has not been found in this clade. Clade 12 contains several species with a clear infective stage, including some typical dauer phenotypes, as well as species without [45]. Because of these inconsistent observations, it is not straightforward to make a claim about dauer in Clade 12. Moreover, the small number of molecular studies regarding dormancy in this clade and their phylogenetic distance to Clade 9 makes direct comparisons complicated.

Nevertheless, we hypothesize that dauer is critical in the evolution of parasitism in Clades 8–12, because it is required to deal with the unpredictability of the presence of a suitable host combined with the nematode’s limited mobility. Here, we will use available knowledge on dauer in *C. elegans* and parasitic nematodes of Clades 9 and 10 as a starting point to discuss this hypothesis. We do this using the definition of a dauer larva as non-ageing, non-feeding, and able to synthesize endogenously a (chemical analogue of) DA to exit the dauer state. Additional morphological characteristics, such as radial constriction and/or a sealed buccal capsule, the ability to survive some degree of desiccation, and adopting a coiled posture, can be part of the dauer state, but are not ubiquitous. To conclude, we will review the dauer-like behaviour in plant-parasitic nematodes of Clade 12, and discuss the experimental data that support the occurrence of a dauer stage in this clade.

Box 1 detailed description of the dauer signalling pathway in *Caenorhabditis elegans* Entry into dauer is regulated by the canonical dauer signalling pathway (Fig. 4), which is initiated by the perception of ascarosides, in particular the dauer pheromone, which is a mixture of specific ascarosides [46, 47]. Ascarosides are species-specific mixtures of 3,6-dideoxy-l-sugar ascarylose derivatives that are excreted and subsequently perceived by nematodes, usually of the same species, in the direct environment. Several different developmental processes are regulated by ascarosides, among which is dauer entry. An increase in the level of dauer pheromone in the environment, consisting of a specific mixture of ascarosides, will result in dauer formation [41, 48–51]. In *C. elegans*, ascarosides are perceived in the sensory neurons of the amphids. Out of 150 discovered ascarosides [52], ascarosides (ascr) #1, #2 and #3 are perceived by GPCRs, SRBC-64 and SRBC-66 [53], whereas ascr#5 is perceived by GPCRs SRG-36 and SRG-37 [54]. Two more GPCRs, dauer formation-37 (DAF-37) and DAF-38, are involved in perception of ascr#2 and ascr#2, 3 and 5, respectively (ref. [55] and Fig. 4). This shows that the perception of different ascarosides is partially overlapping and sometimes redundant between different GPCRs, which suggests the existence of both promiscuous and specific receptors. Indeed, more than 1000 GPCRs are predicted to be present in the genome of *C. elegans* [56, 57], and they play a critical role in the physiological response to a variable environment. When a GPCR in a neuronal cell is activated, it blocks or stimulates, via its G-protein-α subunits, *gpa-2* and *gpa-3*, and the transmembrane guanylyl cyclase DAF-11, the production of the secondary signal cGMP (Fig. 4). This signal targets the α and β subunits of a cyclic nucleotide-gated ion channel, TAX-2 and TAX-4 [58]. Subsequently, the production of insulin-like peptides (INS) and TGF-β is stimulated, and these signals are relayed to the insulin/IGF-1 and TGF-β pathways, respectively, in parallel [57]. The secretion of DAF-28, an INS, is stimulated through ASNA-1, a conserved ATPase. INSs involved in dauer bind to insulin receptor DAF-2, which apart from the dauer signalling pathway also plays a role in ageing, fat metabolism, stress resistance and development [34]. Forty genes encode INSs, of which 19, including DAF-28, have an agonistic effect on DAF-2, preventing dauer. Another 12 INSs have an antagonistic effect on DAF-2, promoting dauer [59]. Through DAF-2, the phosphoinositide-3-kinase (PI3K) AGEing alteration 1 (AGE-1) is activated, and this phosphorylation of phosphatidylinositol 4,5-biphosphate (PIP_2_) into phosphatidylinositol 3,4,5-triphosphate (PIP_3_) in turn activates downstream kinases PDK-1, AKT-1 and AKT-2 [60–62]. AGE-1 is antagonized by DAF-18/PTEN [34]. AKT-1/2 phosphorylate the FoxO transcription factor DAF-16, thereby sequestering it in the cytoplasm (ref. [63] and Fig. 3). Thus, in *daf-2* mutants or INS-antagonized DAF-2, DAF-16 is not phosphorylated and remains in the nucleus. There, it binds promoters, influencing transcription, through its DNA-binding domain (DBD) [34]. One of the players downstream of DAF-16 is AAK-2, a 5′ AMP-activated protein kinase (AMPK), which directs metabolism towards the glyoxylate and gluconeogenesis pathways [19]. Dauer induction by food scarcity is mediated by the insulin pathway, since the perception of food changes the subcellular localization of DAF-16, requiring the insulin-like peptide DAF-28 [64]. Moreover, at this point, temperature cues feed into the insulin/IGF-1 pathway via *ttx-3* [65–67]. Hence, not all environmental cues feed into the pathway at the level of the GPCRs.

Besides INSs, nematodes have two other groups of neuropeptides, called neuropeptide-like peptides (NLP) and FMRFamide-related peptides (FLP). These peptides are derived from precursor peptides, transcribed from neuropeptide-coding genes, and will only acquire their final form after extensive post-translational modifications [68]. Next to INSs, *flp* transcripts likely play a role, since the majority of these genes are upregulated during dauer [69]. Specifically, *flp-10* and *flp-17* play roles in nictation and attraction by CO_2_ [69]. It is not known whether or how FLPs feed into the dauer pathway specifically, but they are expected to be perceived by GPCRs [70, 71].

In parallel to the insulin/IGF-1 pathway, the TGF-β homologue DAF-7 [72, 73] is the cytokine binding to the receptor DAF-1/DAF-4 [74, 75]. Upon ligand binding, the receptor phosphorylates DAF-8 and DAF-14, which inhibits the dauer-inducing transcriptional actions of DAF-3 and DAF-5 in the nucleus (ref. [76] and Fig. 4). Hence, an activated TGF-β pathway favours reproductive development.

The insulin/IGF-1 and TGF-β pathways converge on the activation of DA biosynthesis genes, which produce two steroids, Δ4- and Δ7-dafachronic acid (DA) [35, 36, 77]. DA precursor cholesterol intracellular transport is mediated by *ncr-1* and *ncr-2* [78], and subsequent enzymatic reactions are carried out by Rieske-like oxygenase DAF-36 [79], 3-hydroxysteroid dehydrogenase DHS-16 [80] and cytochrome P450 DAF-9 [35, 81]. DA binds to the nuclear hormone receptor (NHR) DAF-12, resulting in reproductive development [36, 82] by activating transcription of the *let-7* microRNAs. These will, in turn, inhibit *hbl-1*, which stops L2-stage proliferative programs in seam cells, hence dauer induction (ref. [83] and Fig. 4). In the absence of DA, DAF-12 is bound by its corepressor DIN-1, which results in dauer maintenance [84]. The binding of DA, or not, to NHR DAF-12 is the ultimate switch that decides between dauer entry or progression of the life cycle (i.e., reproductive development).

## The dauer stage in parasitic nematodes

Here we will evaluate the phenotypes and underlying molecular mechanisms of dauer stages in zoo- and plant-parasitic nematodes of Clades 8, 9 and 10 and plant-parasitic nematodes of Clade 12. This was done by systematically reviewing the literature, supplemented with the genome information in Fig. 5. To assemble Fig. 5, we used the WormBase ParaSite BioMart orthologue finder tool on a comprehensive list of dauer-related genes from *C. elegans* and queried all available genomes of parasitic nematodes in this database (ref. [85] and Additional file 1: Table S1). Although this tool has its limitations—it will split large gene families into multiple groups, which can result in the false absence of homologues, e.g. for *daf-9*, a cytochrome P450 enzyme, part of a very large gene family with conserved and divergent branches—it does enable one to draw conclusions on clade level. Additional, and more accurate, genomes of parasitic as well as free-living nematode species will further improve this analysis as they become available.

Species in Clade 2 do not harbour the central player involved in DA signalling, *daf-12* (Fig. 5). Although they do show some conserved genes in the TGF-β and insulin/IGF1 pathways, these are not specific to nematodes and therefore not indicative of a dauer stage. Therefore, quiescent stages in this clade, for example in the muscle larvae of *Trichinella spiralis*, even though they bear resemblance to the dauer phenotype, are probably the result of convergent evolution rather than dauer. Therefore, this clade falls outside the scope of this review and will not be discussed further.

With regard to the discussion of dauer in Clades 8, 9, 10 and 12, a word of caution is warranted. For the elucidation of the dauer pathway in *C. elegans*, thorough experimental evidence is available, including genomic, transcriptomic and proteomic studies, and in vivo, in vitro and in silico experiments. *C. elegans* mutants are readily available from the *Caenorhabditis* Genetics Center (University of Minnesota), and there are multiple, easily implementable methods for *C. elegans* transformation available via open access resources such as WormBook. For parasitic nematodes, propagation and genetic tractability are more complicated because of the necessity of a host, which makes the nematode inaccessible during parts of its life cycle [86, 87]. Still, much of the evidence for dauer in parasitic nematodes only consists of genomic and/or transcriptomic data, without in vivo validation. Therefore, we can only assess our hypothesis with caution until more types of functional data become available.

### Dauer in Clade 8 parasitic nematodes

Clade 8 harbours the speciose animal-parasitic orders Ascaridida, Oxyurida and Spirurida and includes zooparasitic species such as *Brugia malayi*, *Toxocara canis* and *Ascaris suum*, as well as invertebrate-associated nematodes and entomopathogens. Many species in this clade are characterized by a life cycle that contains two hosts: an insect as intermediate host and a mammal as the definitive host (Fig. 3). Large parts of the dauer pathway are represented in the genomes of these zooparasitic nematodes (Fig. 5); 55 and 51 dauer pathway genes (out of 64 in *C. elegans*) were identified in *T. canis* and *A. suum*, respectively [88]. However, although the first parasitic *daf-*7 homologue was detected in *B. malayi* [89], this species does not have many genes, especially in the TGF-β pathway, where the R-SMADs and Co-SMADs (*daf-3*, *daf-5*, *daf-8* and *daf-14*) have not been detected, just as in Clade 2. Moreover, only a few *ins* genes are present compared to *C. elegans* (Fig. 5) [88, 90]. By contrast, the DA biosynthetic pathway is largely intact according to the literature [88, 90], *Tc-daf-12* and *Tc-daf-9* are expressed during the infective stage [91], and the presence of endogenous DA was confirmed in both *T. canis* and *A. suum* [88]. Furthermore, a study of *Dirofilaria immitis* (dog heartworm) DAF-12 shows that it can bind DA with remarkable affinity and moreover that dog cholesterol derivatives can bind, albeit with less affinity, to *Dim*-DAF-12 and induce moulting from iL3 into L4 stage [92]. In contrast to these findings, the BioMart tool used to assemble Fig. 5 shows an absence of *daf-9* in nearly all Clade 8 parasites, just as was observed for Clade 9 (Fig. 5). From the presence of endogenous DA we infer that this must be caused by the group splitting inaccuracy of the BioMart tool.

In summary, genomic and biochemical analyses suggest that the DA biosynthetic pathway is conserved in Clade 8 parasites and that it regulates dauer, but this is less clear for the upstream parts of the dauer signalling pathway.

### Dauer in parasitic Clade 9 nematodes

Clade 9 comprises some well-studied zooparasites such as *Haemonchus contortus*, *Nippostrongylus brasiliensis* and *Ancylostoma caninum*. These parasites contain a dauer signalling pathway that allows them to parasitize their hosts [24], sheep, rodents and dog, respectively.

In *A. caninum*, host serum stimulates exit from the infective stage and resumption of reproductive development [44]. Using cGMP signalling chemical activators and inhibitors, it was demonstrated that host cue perception is probably mediated by a GPCR [93, 94]. This receptor is not a homologue of *Ce-srbc-64*, *Ce-srbc-66*, *Ce-srg-36* or *Ce-srg-37*, since these are dauer pheromone (former two) or crowding (latter two) specific [53, 54] and thus free-living or species-specific, respectively. Indeed, they have not been found in genomes of species outside the genus *Caenorhabditis* (ref. [90] and Fig. 5).

Not all dauer pathways start with cGMP signalling, as illustrated by *N. brasiliensis*, a nematode whose infective juveniles only resume development upon a temperature cue. Experiments with chemical inhibitors of specific proteins in the dauer signalling pathway suggest that thermotaxis-induced infective stage exit is not depending on the cGMP signalling pathway, but instead feeds, by unknown means, into the insulin/IGF-1 pathway [95]. Hence, in this nematode, all input for the dauer pathway apart from temperature has been lost. Homologues of the cGMP signalling pathway are still present in the genome (Fig. 5), but might be redundant or have evolved other functions.

Whereas the *C. elegans* genome encodes 40 *ins* genes, most zooparasitic genomes in Clade 9 encode fewer (Fig. 5 and ref. [96]). Experiments with chemical inhibitors and in silico studies suggest that the insulin/IGF-1 pathway is conserved to some extent [95, 97], but more detailed studies demonstrated a discrepancy between *C. elegans* and zooparasitic nematodes. For example, *Hc-age-1* could not rescue *Ce-age-1* mutants [98], and expression patterns of *Hc-age-1* and *Hc-pdk-1* only partially overlap with their homologues in *C. elegans* [99, 100]. The pivotal *daf-16* is conserved (Fig. 5) but has fewer splice variants in *H. contortus* and *A. caninum* than *Ce-daf-16* [96, 101]. In short, the insulin/IGF-1 pathway is roughly conserved but has many diverged details. In particular, the input (INSs) and output (*daf-16* splice variants) seem to have reduced in complexity. This could imply that for these parasites—since dauer is no longer optional such as in free-living nematodes, but rather an obligate stage each juvenile has to pass through—simplification of the pathway has occurred [25], as a decision about entry no longer has to be made.

Components of the TGF-β pathway are less conserved than those of the insulin/IGF-1 pathway [90]. Although most species possess the main players of the TGF-β pathway, their expression profile can be different from or even opposite to that in *C. elegans.* For example, *Ce-daf-7* expression is high in L2 and low in dauer worms, whereas in the zooparasitic nematodes of Clade 9, this is inverted [102–105]. Further downstream, expression levels of *Hc-daf-3* and *Hc-tgfbr2* and their respective homologues *Ce-daf-3* and *Ce-daf-4* show the same opposing trends [106, 107]. Still, *Hc-tgfbr-2* is involved in dauer exit, since RNA interference (RNAi) treatment results in a resumption of development (dauer exit) [107]. These differences in expression profiles have been suggested to be a key trait of parasites that have an obligate requirement to enter an arrested stage [103]. Alternatively, the TGF-β pathway might have adopted a new role in overcoming immune responses from the host [108].

Further downstream, in the steroid signalling pathway, more conservation is observed, since important players such as *daf-12* and *daf-9* have homologues in all species of Clade 9 that were sequenced up to now [90], although not all *daf-9* homologues were detected in our metadata analysis (Fig. 5). The BioMart algorithm breaks down large gene families into groups of 1500 genes. Since the CYP450 gene family, to which *daf-9* belongs, is indeed large, homologues of *daf-9* of distantly related species might be undetected erroneously. Indeed, in several of the species where we did not identify a *daf-*9 orthologue, endogenous DA has been detected (e.g. ref. [105]). Moreover, DAF-12 of *A. caninum*, *A. ceylanicum* and *N. brasiliensis* bind to DA, and this stimulates resumption of feeding [105, 109], which further supports a conserved DAF-9-DA-DAF-12 pathway. As a result, DA has been suggested as a potential anthelmintic drug, since it can force iL3 larvae into reproductive development in the absence of a host [110].

As an alternative ligand for DAF-12, bile acids originating from the host have been suggested to bind to zooparasite DAF-12 and hence integrate host cues into the dauer pathway [110]. Although this hypothesis presents an attractive model for the integration of host cues into the dauer pathway, treatment with mammalian-derived cholestenoic acid did not result in resumption of feeding in *A. ceylanicum* [110], while, by contrast, treatment with DA does, in multiple species [105, 109, 111]. On top of that, *daf-9*, which encodes the last biosynthetic enzyme for the production of DA in *C. elegans*, has since been shown to be present in all zooparasitic genomes studied so far (Fig. 5 and ref. [87]). Moreover, chemical inhibition of *Hc-*DAF-9 prevents recovery from developmental arrest [111], suggesting that zooparasites synthesize an endogenous ligand for DAF-12. Indeed, earlier studies showed that host sterol molecules feed into the dauer pathway upstream of the insulin pathway, rather than into the DA biosynthesis pathway [65, 97, 112].

In conclusion, these observations show conservation of the DA biosynthetic pathway in zooparasitic nematodes of Clade 9. Furthermore, there are indications that host cues can feed into the dauer pathway upstream of the DA biosynthesis and IGF-1/insulin pathways. Conservation of the other parts of the dauer signalling pathway are less convincing, because of the lack of mutant or biochemical evidence. Moreover, the transcriptional regulation of the TGF-β pathway is opposite of that in *C. elegans*.

### Dauer in Clade 10 parasitic nematodes

In Clade 10, a spectrum of more complex life cycles is observed, ranging from facultative to obligate parasitic, using plants, animals and insects as hosts, and with endo- and ectoparasitic lifestyles. Some well-studied species are *Bursaphelenchus xylophilus* (pine wood nematode), *Strongyloides stercoralis* (human pathogen causing strongyloidiasis) and *Parastrongyloides trichosuri* (mammalian pathogen). The fact that many of the parasitic species of Clade 10 can be reared in the lab, independent of a host, or in the case of *B. xylophilus* on an alternative host (fungus), has contributed significantly to the thorough studies of the dauer signalling pathway in these species. In general, these parasites show polyphenic development, which means they switch between parasitic and free-living life cycles depending on host availability. For example, *S. stercoralis* and *P. trichosuri* alternate parasitic generations and free-living generations [28]. L1 larvae, parthenogenetically produced by parasitic females, are excreted in faeces. These juveniles can either immediately become infective larvae, and are developmentally arrested until entering a host, or become a free-living generation feeding on bacteria, in which both males and females exist (Fig. 3). In *S. stercoralis*, post-free-living larvae (larvae whose parent was free-living) are obliged to enter the infective stage dormancy until entering a host, whereas in *P. trichosuri* multiple consecutive free-living generations may occur [1, 113].

The larval stage in which the quiescence occurs is not always L3; *Bursaphelenchus xylophilus*, for example, has not one but two dispersal stages: one (D3) adapted to navigate towards its insect vector and survive winter, which qualifies it as a diapause rather than quiescence, and one (D4) that perceives a host (pine tree) cue upon which it exits its vector and resumes its reproductive development (Fig. 3). The nematode enters these stages during dispersal by their vector, pine sawyer beetles of the genus *Monochamus*. In addition to pine wood, they are able to feed on fungi in dead and dying wood.

The involvement of dauer in the *B. xylophilus* and *S. stercoralis* life cycles has been studied extensively. Interestingly, the development into or from the infective stage is not only influenced by mammalian host cues. In *B. xylophilus*, fatty acid ethyl esters (FAEEs) originating from their vector beetles stimulate the nematode to enter the first dispersal stage, D3 [114]. Additionally, monoterpenes, such as β-myrcene and β-pinene, from the pine tree cause the nematodes to exit their beetle vector and to resume their development [115]. Moreover, D3 larvae are attracted by CO_2_, whereas D4 larvae show avoidance behaviour towards CO_2_, which reveals another means to enter and exit the CO_2_-producing insect vector at the right moment [116]. A pheromone that induces infective stage development was identified in both *P. trichosuri* and *B. xylophilus* [113, 117]. These observations demonstrate that the dauer signalling pathway can integrate multiple biotic and abiotic cues.

In *S. stercoralis*, 85 chemosensory GPCR genes have been identified, of which the majority are expressed in iL3 or L3 [118], underlining the importance of the integration of environmental conditions into development during and after this stage. At least some of these GPCRs are believed to perceive biochemical host cues, relaying the signal to exit the iL3 stage by the secondary messenger cGMP [118]. Furthermore, in *S. stercoralis*, the Gα subunits binding to GPCRs, are present (Fig. 5 and ref. [119]). In *C. elegans*, cGMP is perceived by, among others, TAX-4, before downstream signalling activates the TGF-β and insulin/IGF-1 pathways. When *tax-4* was mutated in *S. stercoralis*, thermotaxis, necessary for localization and penetration of the host skin, was abrogated [120]. *Bursaphelenchus xylophilus* is similarly dependent on temperature, since their vectors are inactive in winter. Hence, the nematodes need to arrest their development in winter until spring. Indeed, thermotaxis in this species is also dependent on *Bxy-tax-4* and, in addition, on *Bxy-tax-2* and *Bxy-daf-11*, as demonstrated by silencing of these genes with RNAi that caused a reduction of the usually long life span under low temperature [13].

In the insulin/IGF-1 pathway, *aap-1*, *age-1*, *pdk-1* and *daf-16* sequences are conserved (Fig. 5) and there is a global overlap in transcriptional patterns [121, 122], although in *B. xylophilus*, dauer gene transcription is divided over its two dispersal stages (D3 and D4) [114, 122]. Chemical inhibition studies of *age-1* in *S. stercoralis* and *B. xylophilus* confirmed its conserved role in dauer regulation [114, 121], and *Ss-daf-16* rescues *Ce-daf-16* mutants [123]. In short, there is a considerable amount of conservation of the insulin/IGF-1 pathway of the Clade 10 parasites.

In the TGF-β pathway, existing knowledge of Clade 10 nematodes largely confirms what is seen in Clade 9 parasites. In *S. stercoralis*, *S. ratti* and *P. trichosuri*, *daf-7* expression is high in the infective stage and low in all other stages [124, 125]. In *B. xylophilus*, *daf-7* expression is upregulated in D3 but not in D4 [114].

A putative homologue of *daf-9* was found in *S. stercoralis* [126] and in *B. xylophilus* [127]. Expression of the latter is very low in the D3 stage and increases in the D4 stage [127], which is expected since upregulation of *daf-9* signals an exit from the dispersal stage. Furthermore, the cytochrome P450 inhibitor ketoconazole inhibits resumption of feeding in *S. stercoralis*, implying that endogenous DA is produced by Ss-DAF-9 [126]. Ss*-*DAF*-*12 is one of the most well-characterized proteins of all parasitic nematodes, and its crystal structure has been resolved [109]. It binds DA, and exogenous treatment with DA leads to prevention of or exit from the infective stage [126]. In the closely related species *S. papillosus*, high DA concentrations prevent the entry into the infective stage [128]. In addition, it was shown that reduced activity of *S. ratti daf-12* impaired the formation of infective larvae and reduced the severity of infection [129]. Interestingly, according to Stoltzfus et al. [118], in *Strongyloides rattii* the insulin pathway acts *downstream* of the steroid biosynthesis pathway, because upon treatment of iL3s with DA, transcription of *ins*s is upregulated. Alternatively, these results could also be explained by a positive feedback loop from the DA biosynthesis pathway to the insulin/IGF-1 pathway. This issue has not been touched upon in other species. In *B. xylophilus*, *daf-12* is upregulated in D3 and D4, showing that it is active when the decision to enter or exit the dispersal stage needs to be made [114, 122, 127]. Furthermore, FAEEs from the vector beetle downregulate DA levels, thereby preventing reproductive development and stimulating entry into the dispersal stages, while exogenous DA treatment blocks entry into the dispersal stages [114].

In conclusion, parasites of Clade 10 have been thoroughly studied with regard to dauer/dispersal stages. Genes involved in thermotaxis are conserved, just as *daf-16* in the insulin/IGF-1 pathway and *daf-12* in the DA pathway. Intriguingly, independently from the type of host or vector (insect, mammalian or plant), these nematodes can integrate host cues into developmental decisions. The similarities between Clade 9 and Clade 10 with regard to changes in the TGF-β signalling pathway suggest that it diverged more from the free-living nematodes than the other parts of the dauer signalling pathway.

### Dauer in Clade 12 parasitic nematodes

Clade 12 harbours both facultative and obligate plant parasites as well as insect parasites. Most attention has been paid to a number of strictly phytoparasitic nematodes such as members of the genera *Pratylenchus*, *Ditylenchus*, *Rotylenchus*, *Radopholus*, *Meloidogyne*, *Globodera* and *Heterodera*. Worldwide agricultural losses due to plant-parasitic nematodes are significant, and seven out of 10 of the economically most destructive plant-parasitic nematodes originate from this clade [130]. In Clade 12, many species are ectoparasites, and hence feed mostly from superficial plant tissues. These species can be compared to, and are sometimes hard to distinguish from, free-living fungivorous species, and they are migratory in all stages [131]. These ectoparasites do not moult into the next stage unless they can feed, and hence they do not survive long without a host.

However, other species of Clade 12 show a clear quiescent stage. For example, the migratory endoparasites of the genera *Ditylenchus*, *Anguina* and *Pratylenchus* are infective during most of their life cycle from the moment of hatching onwards, a common characteristic of migratory endoparasites. When adverse conditions arise, such as absence of a host, low or high temperature, or drought, these species can enter a quiescent state: *Radopholus similis* can survive up to 6 months in absence of a host [132], *Anguina* species can survive extreme temperatures [133] and *Ditylenchus* can enter anhydrobiosis, in which it can survive desiccation for months up to years. A more specific quiescence is present in *Ditylenchus dipsaci*, where under adverse conditions J4 larvae halt development. The arrested J4s are morphologically different from juveniles under optimal conditions; the former have more lipid reserves, are larger and tend to aggregate [12], which are all also characteristics of the *C. elegans* dauer larvae [16]. However, few molecular genetic studies have been done on anhydrobiosis or, more specifically, the quiescence in the J4 stage of *D. dipsaci*; thus molecular overlap with the *C. elegans* dauer pathway is unresolved, and convergent evolution cannot be excluded [40].

In the sedentary endoparasites like root-knot and cyst nematodes, there is a specific infective stage (parasitic J2). For example, the unhatched pre-parasitic J2 (pre-J2) can stay inside their egg, dormant in the soil for long periods of time, up to decades for cyst nematodes of the genus *Globodera* [134], only to hatch when a suitable cue, such as from the host, is perceived. The juveniles hatch as J2, at which point they are morphologically similar to the *C. elegans* dauer stage, with a thickened (although not double) cuticle and radially constricted shape [135]. Additionally, in the potato cyst nematode (PCN), *Globodera rostochiensis*, changes occur in the lipophilicity of the cuticle upon hatching under the influence of potato root exudate [136], similar to those in *C. elegans* larvae exiting dauer [137]. Moreover, they rely heavily on fat deposits as an energy source while they are in the pre- and par-J2 (pre-parasitic and parasitic juvenile) phases [138, 139], just as *C. elegans* dauer larvae [140] and soybean cyst nematode, *Heterodera glycines*, J2 larvae are resistant against toxins [141]. Once hatched, the par-J2 have to locate and enter their host within days in order to establish a feeding site, or they will die (Fig. 2b); they are no longer able to survive an unfavourable environment for an extended period of time.

Therefore, hatching in cyst nematodes needs to be timed in accordance with host presence, i.e. in response to a chemical cue from the host. Subsequently, they need to navigate towards their host, probably using CO_2_ as cue and chemotaxis, in response to chemicals emitted by their host root, such as volatiles, that act as long-distance cues and/or water-soluble compounds that act as more local cues [142]. The sensitivity towards host chemicals for hatching varies considerably. *Meloidogyne* eggs hatch spontaneously upon abiotic cues, such as hydration or temperature increase, and do not require host cues, which might be related to their unusual broad host range. By contrast, soybean and beet cyst nematode (*H. glycines* and *H. schachtii*) hatching is stimulated by host-specific root exudates, but also by abiotic cues such as hydration [143]. On the other side of the spectrum, the PCNs, *Globodera pallida* and *G. rostochiensis*, show little spontaneous hatching by hydration and require a specific chemical stimulus present in potato and tomato root exudates [143]. Indeed, these PCNs have a very narrow host range. *Heterodera glycines*, *G. pallida* and *G. rostochiensis* require triterpenoid hatching factors, coined eclepins. Glycinoeclepin A, B and C have been isolated from soybean and kidney bean, both *H. glycines* hosts, and these induce hatching of *H. glycines* [144], whereas solanoeclepin A has been identified in potato root exudate and induces hatching in *G. pallida*, *G. rostochiensis*, *G. ellingtonae* and *G. tabacum* [145, 146].

All molecular studies on possible dauer-like signalling pathways in Clade 12 parasitic nematodes were done on the phylogenetically distally positioned genera *Meloidogyne*, *Globodera* and *Heterodera*, except for one study conducted in *Radopholus similis*. Transcriptional analysis of juveniles of the latter species displayed upregulation of several genes in the insulin and DA biosynthesis pathways compared to other stages [147], suggesting that the dauer signalling pathway plays a role in the quiescence of this migratory endoparasite.

In *Meloidogyne hapla*, 14 strong and three weak orthologues of 20 *C. elegans* dauer pathway genes have been reported [148]. Expression studies in *Meloidogyne incognita* showed that hatched J2 direct their metabolism towards the glyoxylate pathway, which produces carbohydrates from storage lipids. The temperature- or hydration-induced hatching in *Meloidogyne* spp. shows resemblance to the temperature-induced recovery from the infective stage in *N. brasiliensis* of Clade 9. As described above, in this zooparasite, a temperature cue feeds into the dauer signalling pathway after the cGMP pathway, but before the insulin pathway [95]. Although hatching of *Meloidogyne* spp. is induced primarily by abiotic factors, chemotaxis post-hatching likely involves a cGMP-mediated pathway [149]. Interestingly, several genes of this pathway, such as *gpa-2*, *gpa-3*, *daf-11*, *daf-21* and *tax-4*, seem to have undergone multiple duplications, which has not been detected in any other nematode species (Fig. 5). In *M. incognita*, too, parts of a cGMP signalling pathway have been identified, and functional characterization of *Mi-tax-2* and *Mi-tax-4*—in Clade 10 parasites, such as *S. stercoralis* and *B. xylophilus*, involved in perception of temperature signals and thus involved in the relay of the dauer cue—by RNAi showed behavioural defects. These were observed particularly in host detection, and knockdown resulted in perturbed chemotaxis towards chemicals such as ascarosides [149].

Chemotaxis also occurs in the cyst nematodes [150]. FMRF-amide-like peptides (FLPs) are responsible for locomotion [151], so they could be responsible for initiation of movement after hatching in *G. pallida*, marking the activation of the dauer exit pathway in order to exploit a suitable food source, and ultimately, redirect development towards reproduction. Indeed, the expression of *flp*s is upregulated in the *H. avenae* [135] and *G. pallida* [69] par-J2 stage, just as in *C. elegans* dauer [69]. The involvement of *flp*s in locomotion associated with dauer is further supported by the fact that downregulation of *flp*s in the closely related root-knot nematode species *M. incognita* and *M. graminicola* reduces hatching and infectivity [152–155].

Information on conservation of the downstream parts of the dauer pathway in cyst and root-knot nematodes is scarce. However, the insulin pathway seems to be conserved in *Meloidogyne* spp., since many homologues, including the pivotal *daf-2* and *daf-16*, were detected in the *M. incognita* and *M. hapla* genomes. Downregulation by RNAi of *Mi-daf-16* and *Mi-skn-*1, encoding a transcription factor acting parallel to DAF-16, reduced the hatching rate, infectivity, growth and reproduction rate [156]. Additionally, players in the DA signalling pathway such as *daf-9* and *daf-12* were identified [90, 157]. For the majority of species, the pivotal *daf-12* gene was detected in the genome (Fig. 5). *Mi-nhr-48*, of which the homologue in *C. elegans* is the closest paralogue to *daf-12*, is essential for pathogenicity, but a connection to the dauer pathway was not made [140]. In conclusion, apart from bioinformatics studies, there is little functional evidence for a dauer pathway in the Clade 12 plant-parasitic species.

Indeed, several comparative genomics [158, 159] and transcriptomics [135, 159] studies conclude that there is no overlap between dauer signalling and hatching followed by host-finding behaviour of cyst and root-knot nematodes. For example, transcriptional patterns in hatched J2 of *H. glycines* did not match those of *C. elegans* dauers [159], and only a few homologues of dauer pathway genes were found in the *G. pallida* genome, and treatment with cGMP unexpectedly inhibited hatching in quiescent nematodes under the influence of potato root diffusate (PRD) [158]. However, below we provide arguments why these results do not necessarily refute the hypothesis that dauer is involved in the infectious stages of these plant-parasitic nematodes. Firstly, the timing of sampling is crucial, since it is not clear what marks the end point of quiescence in cyst nematodes: the hatch of J2 (i.e., resumption of nematode movement), or the resumption of feeding and the subsequent moult from J2 to J3 inside the host. On the one hand, hatching marks an increase in mobility and an end to the stress-resistant and non-feeding long-lasting survival stage. On the other hand, infective stages of parasites usually end when the parasite has entered its host and moults into the next larval stage, and moreover, motile J2 in search of the host plant are the functional equivalent of the iL3 stage of zooparasitic species. It is not clear, and difficult to ascertain, which of these options is most likely, and the transcript level of dauer signalling pathway genes should be evaluated in order to provide clarity. Secondly, sampling of large numbers of J3 nematodes is not trivial, since by then the larvae are inside the host. The same problem holds for sampling of J1 juveniles, because they are mostly present in immature cysts, attached to the plant root. Hence, making a detailed comparison between J1, before developmental arrest, J2, still in arrest, and J3, which just resumed development, is difficult. The abovementioned studies do not compare these critical stages, and relevant dauer up- or downregulated genes are therefore likely to remain concealed. Thirdly, the currently available genome sequences of many Clade 12 nematodes are highly fragmented, which will result in incomplete read mapping and thus an incomplete analysis of differentially expressed genes. Finally, because of the large differences in the environments to which *C. elegans* dauers on the one hand, and cyst and root-knot nematodes on the other are exposed, it is highly unlikely that life-stage-specific transcription is similar. For example, a *G. rostochiensis* hatched J2 juvenile resides in the soil and needs to find its host as soon as possible, upon which it needs to be able to penetrate the root. This will require transcription of specific receptors involved in chemotaxis for navigation and plant cell wall-degrading enzymes for root entry. By contrast, the *C. elegans* dauer, which can be found in a range of environments, might nictate but otherwise is mostly immotile and not actively pursuing host cues. Moreover, phytoparasitic nematodes acquired a number of effectors, in part via horizontal gene transfer, which are active in the infective stage [160], and homologues of these genes will not be found in *C. elegans*. Hence, cyst and root-knot nematode infective stages likely display a very different transcriptional pattern from that of *C. elegans* dauer larvae, and other experimental approaches are necessary to prove or refute the hypothesis that dauer signalling is responsible for quiescence in Clade 12 parasites.

## Implications and prospects on dauer in parasitic nematodes

Throughout the phylum Nematoda, numerous parasitic lineages arose independently, and different types of quiescence have evolved since parasites need this feature to synchronize their life cycle with their host [41]. Although in thoroughly studied animal-parasitic nematodes of Clades 9 and 10 the infective juvenile usually is in the third larval stage (iL3), this cannot be generalized to nematodes with other parasitic lifestyles. Within the phylum Nematoda, there are examples of dormancy in any larval stage. For example, Clade 8 *Brugia malayi* arrests in L1 (Fig. 3) and Clade 12 *H. schachtii* arrests in J2. Whereas dormancy occurs throughout the phylum, the dauer stage is specific for Clades 8 to 12. However, in many species, next to an infective stage or dispersal stage, a diapause is observed, which is entered depending on the season, such as in *H. contortus*, [161], *B. xylophilus* [13] and *G. rostochiensis* [162]. When in diapause, nematodes do not respond to host cues. Hence, dauer is only a specific type of hypobiosis, whereas multiple other types of hypobiosis occur throughout the phylum in both parasites and free-living nematodes. However, of these hypobiotic stages, dauer is the most completely molecularly elucidated survival stage, because it is the most prominent form of hypobiosis in the model organism *C. elegans*.

In parasitic nematodes closely related to *C. elegans*, there is overlap in morphological and behavioural aspects of dauer, such as the buccal plug, radial constriction, double cuticle and the ability to survive desiccation. In the dauer-like stages of parasites from different clades (8, 10, 12), these morphological characteristics are less pronounced or absent. This suggests that these aspects of dauer were not selected for in the evolution of parasitism; only the ability to bridge time in food scarcity was [41]. Nevertheless, although the morphological features of the dauer juveniles diverged in these distant clades, the molecular mechanisms underlying this survival trait are likely conserved, as we discuss in the present review.

The GPCRs that perceive dauer pheromone, a mixture of ascarosides, in *C. elegans* are not at all conserved, not even in other *Caenorhabditis* species. This is not surprising, since ascarosides are highly species- and habitat-specific. Additionally, GPCRs are presumed to be involved in chemotaxis and perceiving host cues, but similarity in chemotaxis between species is not explained by phylogeny, but rather by host preference [108]. Indeed, this system is evolutionary easily adaptable and thus can diverge quickly, as illustrated by several accelerated evolution experiments. For example, when worms were grown in high density for multiple generations, their offspring acquired multigenic resistance to crowding-induced dauer because of a disruption in receptor genes *srg-36* and *srg-37* [54]. In another experiment with the polyphenic nematode *P. trichosuri* (Clade 10), it was demonstrated that natural variation in sensitivity to dauer-pheromone-like cues, produced by free-living worms of the same species, exists in the population [113]. Culturing worms in the presence of these pheromones favoured natural selection for free-living generations, and vice versa. The resulting inbred lines were insensitive or supersensitive, respectively, to the pheromone. Hence, these experiments show how adaptations to the dauer pathway could have arisen. Furthermore, it is plausible that the cGMP signalling pathway and its plasticity play an important role in chemotaxis.

In the insulin pathway, a striking expansion of the *ins* gene family has occurred in *C. elegans*, most of which are absent in parasitic nematodes (Fig. 5), and also in other *Caenorhabditis* species [90]. Generally, only the antagonists of insulin receptor DAF-2 encoding genes, *ins-1* and *ins-18*, are widely conserved (Fig. 5 and ref. [90]). It has been suggested that, since no known agonists of DAF-2 are detected in parasitic nematodes, host insulins might serve as a complementary source of agonists for the insulin receptor [163].

The TGF-β pathway seems to be the least conserved part of the dauer signalling pathway. Indeed, our genomics meta-analysis shows that a number of important transcription factors—*daf-3*, *daf-5* and *daf-14*—are not conserved. To a lesser extent, that also holds for *daf-7* that is not present in Clade 12 and a part of Clade 10 (Fig. 5). Moreover, the expression pattern of the pivotal *daf-7* gene in parasites is generally opposite of that in *C. elegans* [102–105, 114, 124, 125]. There have been multiple suggestions to explain this discrepancy; the TGF-β pathway might be adapted to cope with immune responses from the host [108] or has flipped expression patterns since the infective stage is obligatory instead of facultative in many parasites [103]. Gilabert et al. [90] suggested that the high expression of *daf-7* in *C. elegans* dauer is derived, and that in ancestors and in contemporary parasites, DAF-7 is mainly responsible for iL3 to adult transition, hence the high expression in infective juveniles.

The almost ubiquitous presence of some genes, such as *tax-2*, *tax-4*, *daf-1*, *age-1*, *pdk-1* and *daf-16* (Fig. 5), across all clades is not a suggestion that dauer is ubiquitous as well. Rather, these genes are part of the ancient cGMP, TGF-β and insulin/IGF-1 signalling pathways that occur throughout the animal kingdom and hence are not specific for dauer. However, when the group formerly known as Secernentea evolved from Clade 7, it developed a specialized form of quiescence, which is known as dauer, using these ancient pathways to regulate entry and exit. The presence of dauer in the ancestor of Clades 8 to 12 may have enabled the evolution of a dispersal stage, possibly in association with an insect vector. Then, for the evolution into an infective stage in parasites, the pathway may have lost complexity, especially for specialist parasites, as is observed from genomic data in multiple parasitic species in all of the Secernentea clades. Free-living nematodes need to integrate many different cues in order to decide whether or not to enter dauer, depending on what habitat and abiotic conditions they are exposed to. Parasites, on the other hand, have a less variable habitat, since a (large) part of their life cycle occurs in or around their intermediate or definitive host. The presence of these hosts largely determines whether conditions are favourable. For some parasitic nematodes, the infective stage will be entered unconditionally, and the only decision that needs to be made is when the quiescent state is exited. This decision can be made with a cue of low complexity: a molecule originating from the host, or even only a change in abiotic conditions such as temperature or humidity. Therefore, a reduction in dauer pathway complexity is possible. This reduction is visible mostly in GPCRs, complexity of *ins* signalling and the lack of conservation of the TGF-β pathway (Fig. 5). These genes, although still crucial, have reduced in number possibly because a high degree of plasticity is not necessary for parasites because their habitat is less variable than that of free-living nematodes.

It is widely accepted—and substantiated in this review—that many nematodes of Clades 9 and 10 possess a dauer stage—also called infective stage. Despite their phylogenetic distance, we postulate that this analogy can be extended to other parasitic nematodes, such as in Clade 12, even though the occurrence of dauer per se in this group has not been unambiguously demonstrated. A likely explanation for this is the large phylogenetic distance to the model organism *C. elegans*, which hampers identification of similarities between the *C. elegans* dauer pathway and infective stage pathways of these plant-parasitic nematodes. However, in many of the Clade 12 economically relevant parasites such as the root-knot and cyst nematodes, hatching and subsequent host-finding by chemotaxis are the most pivotal events in infection [142]. These processes rely on host chemicals, which likely feed into the dauer signalling pathway, and the morphology and behaviour of infective J2 larvae loosely resemble dauer worms of parasites of Clade 9 and 10. Moreover, many homologues of dauer pathway genes are present in the genomes of these species (Fig. 5), despite the as yet suboptimal quality of these genome sequences. However, apart from bioinformatics studies, the experimental evidence is scarce. Thus, although it seems likely that quiescence in Clade 12 is originating from the dauer signalling pathway based on genomics data, functional studies are required to substantiate this hypothesis.

Interestingly, it was suggested that host cues might directly bind to DAF-12 of parasites of Clades 9 and 10 in order to release dauer: mammalian bile acid hormones would bind the *A. ceylanicum* and *D. immitis* DAF-12 [92, 110]. This is a relevant possibility for Clade 12 parasites too, and indeed it was speculated that eclepins may directly activate *Globodera* and *Heterodera* homologues of DAF-12 [164], which would support involvement of the dauer pathway in quiescence in cyst nematodes. This would also explain the absence of *daf-9* homologues in numerous species (Fig. 5), although we deem it more likely that the BioMART algorithm does not work optimally for the large gene family of the CYP450s, and that hence the absence of *daf-9* is erroneous. Indeed, although in zooparasites some host-derived bile acid derivatives can bind to parasitic DAF-12 in vitro, their binding is weaker than that of DA itself, and in vivo experiments show that only the latter is able to promote exit from the infective stage [92, 110].

An alternative hypothesis is that host cues feed more upstream into the dauer signalling pathway, more specifically, via GPCRs through the cGMP signalling pathway, similar to dauer pheromones of *C. elegans*. Nematodes contain a wide variety of *gpcr* genes, which have evolved to perceive species-specific relevant cues. Some of these might perceive cues that initiate dauer exit in order to release the developmental arrest. Indeed, even in nematodes outside Clade 9, such as *S. stercoralis* of Clade 10, DA and the biosynthetic genes, such as *daf-9*, are still present [126]. Similarly, *daf-*9 homologues are detected in Clade 12 parasites, albeit not in all species (Fig. 5). Therefore, it is likely that DA is endogenously produced and not (or not completely) substituted for by host-produced molecules. Secondly, a host signal feeding into the pathway via a GPCR opens the possibility for modification of the signal more downstream, allowing the dauer pathway to integrate several different cues of different origins, thereby retaining the flexibility of the pathway as seen in *C. elegans*. For example, for *B. xylophilus* and *Heterodera* and *Globodera* spp., these other cues could be temperature, since they can enter a seasonal diapause, which makes them insensitive to host cues [13, 158]. In conclusion, if the pre-parasitic and/or parasitic J2 stage of cyst nematodes is a state analogous to dauer, then the eclepins are the signal to exit from this dauer-like stage, but it is as yet unknown how these cues are integrated into the dauer pathway of these species. A better understanding of the mechanism behind the integration of these host-derived cues into the dauer signalling pathway could facilitate the development of synthetic analogues that can induce ‘suicide hatching’ and thereby serve as a specific and effective new control method [165]. Analogous to the plant parasites, similar host cue analogues for zooparasites could reduce their infectivity in humans and cattle. However, both of the above-described hypotheses—direct binding of host cues to DAF-12 and entry of the host cue more upstream into the dauer pathway—are not sufficiently supported by evidence and need to be experimentally confirmed. Although there is a lack of simple transformation tools for plant-parasitic nematodes, many other tools to study the dauer signalling pathway have already proven useful in zooparasites, such as *C. elegans* complementation studies (e.g. [98]), functional assays with heterologously produced homologues of dauer pathway proteins (e.g. [105]) and in vitro receptor-ligand interaction assays (e.g. [83]).

## Conclusions

Whereas zooparasitic nematologists have coined the term ‘dauer’ to explain a very specific type of quiescence in free-living nematodes such as *C. elegans*, and use the term ‘infective stage’ for derived stages in parasites, the plant-parasitic nematology community mostly uses ‘dauer’ as a phenotypic trait related to survival under environmental stress and/or food shortage without a confirmed link to an underlying molecular mechanism. We propose here to use dauer only for the non-ageing, non-feeding state, whose entrance and/or exit is regulated by the DA pathway—specifically, by the endogenous production of DA. Phenotypic features and -omics data suggest that the dauer hypothesis can be expanded to include the plant-parasitic nematodes of Clade 12. However, Clade 12 parasitic nematodes display much more variation in dauer-like quiescence than those from Clades 9 and 10. This is perhaps not unexpected, since members of this clade are only distantly related to the best described dauer model, *C. elegans*. The phenotypic and molecular diversification complicates a straightforward identification of similarities between the clades. After Clades 8 to 12 arose from Clade 7 and the first dauer-like stage evolved, the ancient cGMP, TGF-β and insulin/IGF-1 pathways were co-opted to regulate this new dauer signalling pathway. The DA biosynthetic pathway, on which these three ancient pathways converge, is specific for Clades 8 to 12, and thus has evolved after this divergence. In all clades harbouring nematodes with a parasitic lifestyle, host-derived chemical cues influence the quiescence of parasitic nematode species. There is a large body of evidence showing that these cues feed into the dauer signalling pathway, either into the DA pathway or through the GPCRs. We postulate that this is also the case for host cues of the Clade 12 plant-parasitic nematodes.

## Supplementary Information

**Additional file 1: Table S1.** The accession numbers of homologues in parasitic nematodes of *C. elegans* dauer signalling pathway genes, as detected using the WormBase ParaSite (release number 14.0) BioMart orthologue finder tool (ref. [85], https://parasite.wormbase.org) and that was the basis for the construction of the heat map in Fig. 5. If multiple homologues were detected, they are ordered from high to low % identity.

## Data Availability

The datasets analysed during the current study are available in the BioMart WormBase ParaSite repository, https://parasite.wormbase.org/info/Tools/biomart.html.

## Abbreviations

age: AGEing alteration
ascr: Ascaroside
cGMP: Cyclic guanosine monophosphate
CYP450: Cytochrome P450
D3, D4: Dispersal stage 3 and 4
DA: Dafachronic acid
daf: Dauer formation
FLP: FMRFamide-related peptides
gpa: G-protein-α subunit
GPCR: G-protein-coupled receptor
IGF-1: Insulin growth factor 1
iL3: Infective larval stage 3
ins: Insulin-like peptide
J1, J2, J3, J4: Juvenile stages 1, 2, 3 and 4
L1, L2, L3, L4: Larval stages 1, 2, 3 and 4
L2d: Pre-dauer larval stage 2
NHR: Nuclear hormone receptor
NLP: Neuropeptide-like peptide
par-J2: Parasitic J2
PCN: Potato cyst nematode
PI3K: Phosphoinositide-3-kinase
PIP_2_: Phosphatidylinositol 4,5-biphosphate
PIP_3_: Phosphatidylinositol 3,4,5-triphosphate
pre-J2: Pre-parasitic J2
TGF-β: Transforming growth factor beta
